# Assessing the relationship between physical activity and the gut microbiome in a large, population-based sample of Wisconsin adults

**DOI:** 10.1371/journal.pone.0276684

**Published:** 2022-10-26

**Authors:** Elizabeth A. Holzhausen, Kristen C. Malecki, Ajay K. Sethi, Ronald Gangnon, Lisa Cadmus-Bertram, Courtney L. Deblois, Garret Suen, Nasia Safdar, Paul E. Peppard

**Affiliations:** 1 Department of Integrative Physiology, University of Colorado-Boulder, Boulder, Colorado, United States of America; 2 Population Health Sciences, School of Medicine and Public Health, University of Wisconsin-Madison, Madison, Wisconsin, United States of America; 3 Department of Bacteriology, University of Wisconsin-Madison, Madison, Wisconsin, United States of America; 4 Microbiology Doctoral Training Program, University of Wisconsin-Madison, Madison, Wisconsin, United States of America; 5 Department of Medicine, University of Wisconsin-Madison, Madison, Wisconsin, United States of America; 6 The William S. Middleton Memorial Veterans Hospital, Madison, Wisconsin, United States of America; University of Thessaly, GREECE

## Abstract

The gut microbiome is an important factor in human health and disease. While preliminary studies have found some evidence that physical activity is associated with gut microbiome richness, diversity, and composition, this relationship is not fully understood and has not been previously characterized in a large, population-based cohort. In this study, we estimated the association between several measures of physical activity and the gut microbiota in a cohort of 720 Wisconsin residents. Our sample had a mean age of 55 years (range: 18, 94), was 42% male, and 83% of participants self-identified as White. Gut microbial composition was assessed using gene sequencing of the V3-V4 region of 16S rRNA extracted from stool. We found that an increase of one standard deviation in weekly minutes spent in active transportation was associated with an increase in alpha diversity, particularly in Chao1’s richness (7.57, 95% CI: 2.55, 12.59) and Shannon’s diversity (0.04, 95% CI: 0.0008, 0.09). We identified interactions in the association between Inverse Simpson’s diversity and physical activity, wherein active transportation for individuals living in a rural environment was associated with additional increases in diversity (4.69, 95% CI: 1.64, 7.73). We also conducted several permutational ANOVAs (PERMANOVA) and negative binomial regression analyses to estimate the relationship between physical activity and microbiome composition. We found that being physically active and increased physical activity time were associated with increased abundance of bacteria in the family Erysipelotrichaceae. Active transportation was associated with increased abundance of bacteria in the genus *Phascolarctobacterium*, and decreased abundance of *Clostridium*. Minutes in active transportation was associated with a decreased abundance of the family Clostridiaceae.

## Introduction

The human gut microbiome is home to more than 1,000 unique species of microbes [1] and plays an important role in host physiology, metabolism, nutrition, and immune system development and maintenance [2]. Microbiome dysbioses have been associated with irritable bowel disease [3], Crohn’s disease [4], Type 1 and Type 2 diabetes [5, 6], asthma and allergy [7, 8], and rheumatoid arthritis [9]. Moreover, the gut microbiome has been linked to obesity [10, 11], colorectal cancer [12], cardiovascular disease [13], autism [14, 15], and stress, anxiety, and depression [16]. Because of the vital contributions that gut microbes make to human health, it is important to understand how certain exposures may improve gut health.

Physical activity is a critical component of human health and may alter the health of the gut microbiome. In particular, physical activity has been associated with reduced adiposity, reduced mortality, and improved cardiometabolic health [17]. The relationship between physical activity and the microbiome has been well-established in animal models [18–22]. In humans, the relationship is less well understood. Previous studies that compared athletes with sedentary controls have found that athletes have increased alpha-diversity and enriched functional pathways associated with host health, including amino acid synthesis, carbohydrate metabolism, and short-chain fatty acid synthesis [23, 24]. Acute changes have also been observed in athletes during intense activity [25–27]. While these studies indicate a possible relationship between physical activity and the gut microbiota, they represent extreme physical activity conditions, relative to people with more common physical activity habits [28].

Several studies have been conducted in more general populations, but results have been mixed. Some have found that increased physical activity/fitness is associated with increased alpha-diversity [29], increases in butyrate-producing taxa including *Clostridiales*, *Roseburia*, *Lachnospiraceae*, and *Erysipelotrichaceae* [29–31], or shifts in the abundance of other taxa [32, 33], whereas others have not observed a relationship [34]. While these studies shed some light on the possible effect of physical activity on the composition, richness, and diversity of the gut microbiome, they are limited by low sample size, with many of them having fewer than 100 participants [30, 33–35]. Here, we address these limitations by considering a large population with 720 participants and estimate the association between both objective and subjective measures of physical activity and the richness, diversity, and composition of the gut microbiome.

## Materials and methods

### Study population

The data used in this analysis come from the Survey of the Health of Wisconsin (SHOW) and its ancillary microbiome study, Winning the War on Antibiotic Resistance (WARRIOR), whose methods have been described in detail [36–38]. The SHOW and WARRIOR projects were approved by the University of Wisconsin Institutional Review Board (2013–0251). Briefly, SHOW–at its initiation–was an annual cross-sectional survey of a randomly selected, representative sample of Wisconsin residents over the age of 18. Since then, SHOW has expanded to collect longitudinal samples in a subset of adults and comprehensive data on participants’ health and health history, environmental and neighborhood exposures, and several objective physical measures. During 2016 and 2017, as part of the WARRIOR ancillary project, participants also provided stool samples for microbiome analysis and responded to microbiome-specific questionnaires. This analysis uses survey data, microbiome data, and objective measures of physical activity, sedentary time, and sleep data from this study.

Participants were excluded from this analysis if they reported that they were taking antibiotics at the time of stool sample collection; however, those who used antibiotics during the past year were included. Three participants were excluded due to use of antibiotics.

SHOW sampling methods allow for multiple eligible participants per household. The 720 participants who provided high-quality stool samples included in this analysis were from 567 unique households.

### DNA extraction, PCR, and sequencing

The DNA extraction methods used in this study have been described in detail previously [37, 39]. Briefly, chemical, heat, and mechanical methods were used to lyse the bacterial cells found in stool samples. The extracted DNA was purified using a phenol-chloroform-isoamyl wash, followed by NucleoSpin Gel and a PCR clean-up kit (Mcherey-Nagel, Germany). The DNA was quantified using PicoGreen in a microplate reader.

The V3-V4 region of the 16S rRNA genes in the extracted DNA was barcoded and amplified as described in Kozich *et al*. [40]. Following PCR, amplicons were subjected to gel electrophoresis using 1.0% low melt agarose (National Diagnostics, Atlanta, GO). Bands of the approximate amplicon length were extracted and purified using a Zymo gel DNA Recovery Kit (Zymo Research, Irvine, CA, United States). Samples were then quantified using a Qubit^®^ Fluorometer (Invitrogen, San Diego, CA, United States) and pooled to 4nM to construct a sequencing library. Samples were then sequenced on an Illumina MiSeq sequencer (Illumina, Inc., San Diego, CA), using an Illumina MiSeq V2 (2x250bp) Reagent Kit (Illumina, Inc., San Diego, CA) per manufacturer’s instructions.

### 16s rRNAs sequencing and data processing

Raw sequencing data were processed using mothur (v. 1.43.0) [41] following the Standard Operating Procedure for MiSeq data [40]. Briefly, contigs (overlapping sequences) were aligned using the SILVA database (v. 132) [42], low-quality reads and chimeras detected by UCHIME [43] were removed, and sequences were assigned to operational taxonomic units (OTUs) with a threshold of 97% similarity using the GreenGenes database [44]. Rare OTUs, defined as those whose relative abundance was less than 0.001% of the overall OTU count, were removed. Alpha-diversity metrics were calculated using the phyloseq package in R [45].

### Physical activity and objective sleep measures

Participants wore two accelerometers (wGT3xBT—ActiGraph Corporation, Pensacola, FL), one was worn on the hip (to measure physical activity), and the other was worn on the wrist (to measure sleep). Participants were instructed to wear the monitors for seven consecutive days, except during activities that could get the monitor wet, like swimming, showering, or bathing. The hip accelerometer was worn during waking hours only; the wrist accelerometer was worn continuously for 24 hours/day. Data were aggregated into 60 second epochs for scoring and validation.

Hourly data were collected from both the wrist and hip accelerometers and aggregated into six-hour intervals. Of the 720 participants, 625 agreed to participate and contributed at least 1 hour of accelerometry data. The remaining 95 participants did not contribute any accelerometry data. S1 Fig summarizes the intervals with missing data prior to imputation. Because all missing data were imputed, we did not exclude any participants with low wear time. Use of imputation has been shown to be less biased and more precise, compared to methods that discard observations with low wear time [46].

We labeled the period between midnight to 5:59 AM as “night”, 6 AM to 11:59 AM as “morning”, 12 PM to 5:59 PM as “afternoon”, and 6 PM to midnight as “evening”. After aggregation, intervals that did not include at least 300 minutes of wear time out of the possible 360 minutes of measurement were set to missing (and later imputed), while intervals with 300 or more minutes of wear time were scaled up to the full 360 minutes.

### Wrist accelerometer measures

Sleep duration was measured via wrist accelerometer. Wrist accelerometer data were aggregated into 60-second epochs for validation, scoring, and analysis. Sleep data were scored both manually and automatically, with in-bed and out-bed times identified manually, based on activity recorded by the accelerometer in conjunction with paper logs filled out by participants. The Cole-Kripke algorithm was used to distinguish sleeping and waking periods [47].

### Hip accelerometer measures

Physical activity was measured via hip accelerometer. Freedson cut points were used to distinguish between sedentary, light, moderate, and vigorous physical activity levels [48]. Specifically, Freedson cut points categorize accelerometry data, measured in counts per minute, into metabolic equivalents (METs) categories: light (≤ 2.99 METs), moderate (3.00–5.99 METs), and vigorous (≥6.00 METs). Therefore, any activity that was classified as moderate or vigorous (≥ 3.00 METs) by the Freedson cut points was labeled moderate to vigorous physical activity (MVPA). In these analyses, we used accumulated minutes of activity rather than bouted activity, adjusted to a 7-day week.

Participants were classified as physically active if they met the guideline for completing 150 minutes of moderate or vigorous intensity physical activity per week [49].

### Self-reported measures

During the computer assisted personal interview portion of the study, participants were asked, “In a typical week, do you walk or use a bicycle for at least 10 minutes continuously to get to and from places?”. Those who responded yes to this question were classified as participating in active transportation and were asked additional questions to characterize the average time per week that they spent walking or bicycling on a typical day for travel.

### Other information

Participants’ primary self-identified race was used to create the categories used in these analyses: Non-Hispanic White, Non-Hispanic Black, Hispanic, and other. Body mass index was calculated based on measures of weight and height that were collected during an in-home interview by trained staff. Weight was measured using a digital calibrated scale (Health-O-Meter 725 KL–Sunbeam Products, Bridgeview, IL), and height was measured in duplicate using a stadiometer (SECA 222 wall-mounted stadiometer–SECA Corp., Hanover, MD).

Participants self-reported their total household income before taxes in the previous 12 months, as well as the household members (including children) who were supported by that income. Households were classified as urban or rural using the 2010 Census urbanized areas and urban cluster classification [50]. Self-reported education was classified as some high school, high school, some college, bachelor’s, and more than bachelors. Never smoking was defined as not smoking more than 100 cigarettes during their entire lives.

Diabetes status was ascertained via a blood sample. Participants were classified as diabetic if their blood HgbA1c level at the time of participation was greater than or equal to 6.5 or if they self-reported a previous physician diagnosis of Type 1 or Type 2 diabetes. Participants were classified as having depression if they reported any depression symptoms or reported taking antidepressant medications in the past 30 days. Depression symptoms were collected during an Audio-Computer Assisted Self-Interview (ACASI) portion of the home visit, using the Patient Health Questionnaire-2 (PHQ-2) [51]. Participants also reported any medications they took in the past 30 days during the computer assisted personal interview (CAPI) portion of the home visit. This list of medications was compared with the RxNorm database to identify antidepressant medications [52]. Proton pump inhibitor and antibiotic use during the past year were gathered using a self-administered questionnaire.

All dietary intake measures (carbohydrates, fat, protein, fiber, and alcohol) were collected using the National Cancer Institute’s Diet History Questionnaire (DHQ) [53]. Participants with dietary variables above the 99^th^ percentile were windsorized to the 99^th^ percentile to account for the positively skewed distribution and known limitations of the dietary instrument. Finally, although not significantly associated with physical activity, we control for sample age in all analyses. Sample age was the time between when the stool sample was produced and when it was put into storage at -80°, as time spent in cold storage during shipping may impact some microbiome measures used in this analysis [54].

### Data analysis

#### Imputation

With the *mice* package (version 3.14.0) in R, multiple imputation was used to estimate the probable values for all missing data [55]. Predictors were chosen using *mice’s* quickpred function, whose steps have been previously detailed [56].

#### Statistical analysis

To estimate the association of physical activity with alpha-diversity, we generated several linear mixed effects models with random intercepts to account for multiple participants from the same household, using R (version 4.1.2). We used physically active status, MVPA minutes per week, use of active transportation, and minutes per week of active transportation as our primary predictors and Chao1, Shannon, and Inverse Simpson as the outcome variables. We performed bivariate regression analyses to test whether *a priori* possible confounders of the relationship between physical activity and the microbiome were significantly associated with physical activity. Age, race/ethnicity, body mass index, household income, census category, smoking status, diabetes, depression, use of proton pump inhibitors, use of antibiotics, dietary intake of carbohydrates, protein, fat, fiber, and alcohol, sleep duration, sedentary time, and light activity were statistically significantly associated with physical activity (*P* < 0.05) and were included as covariates in our analyses (S1 Table). We additionally included sex and education as covariates. Tests for significant interaction were performed between each outcome variable of interest and each covariate listed above. Significant interactions are presented in the results where applicable. We tested for non-linearity in each of the primary predictor variables but did not find evidence of a non-linear relationship.

Additionally, we generated several non-metric multi-dimensional scaling plots to visualize differences in microbiota composition by physical activity measures, using Bray-Curtis distance matrices calculated by the *vegan* package in R [57]. We then ran a PERMANOVA analysis [58] on the distance matrices to examine the association between physical activity and bacterial community composition (n = 1,000 permutations per run). Imputed data was used for these calculations but was unable to be pooled. One imputation was used for each calculation, rather than all imputations pooled together. Each analysis was run on several different imputations to confirm that the results were robust to the choice of imputed data set.

Next, we used negative binomial regression on the raw counts of individual genera and families to estimate the relationship between the abundance of individual taxa and physical activity. To reduce multiple testing and the effect of zero-inflation in our data set, we removed taxa that were comprised of more than 30% zeros. An offset of the log of total reads was used, and all models were adjusted for important covariates. Analyses were adjusted for multiple testing using the Benjamini-Hochberg procedure [59].

## Results

**Fig 1:**
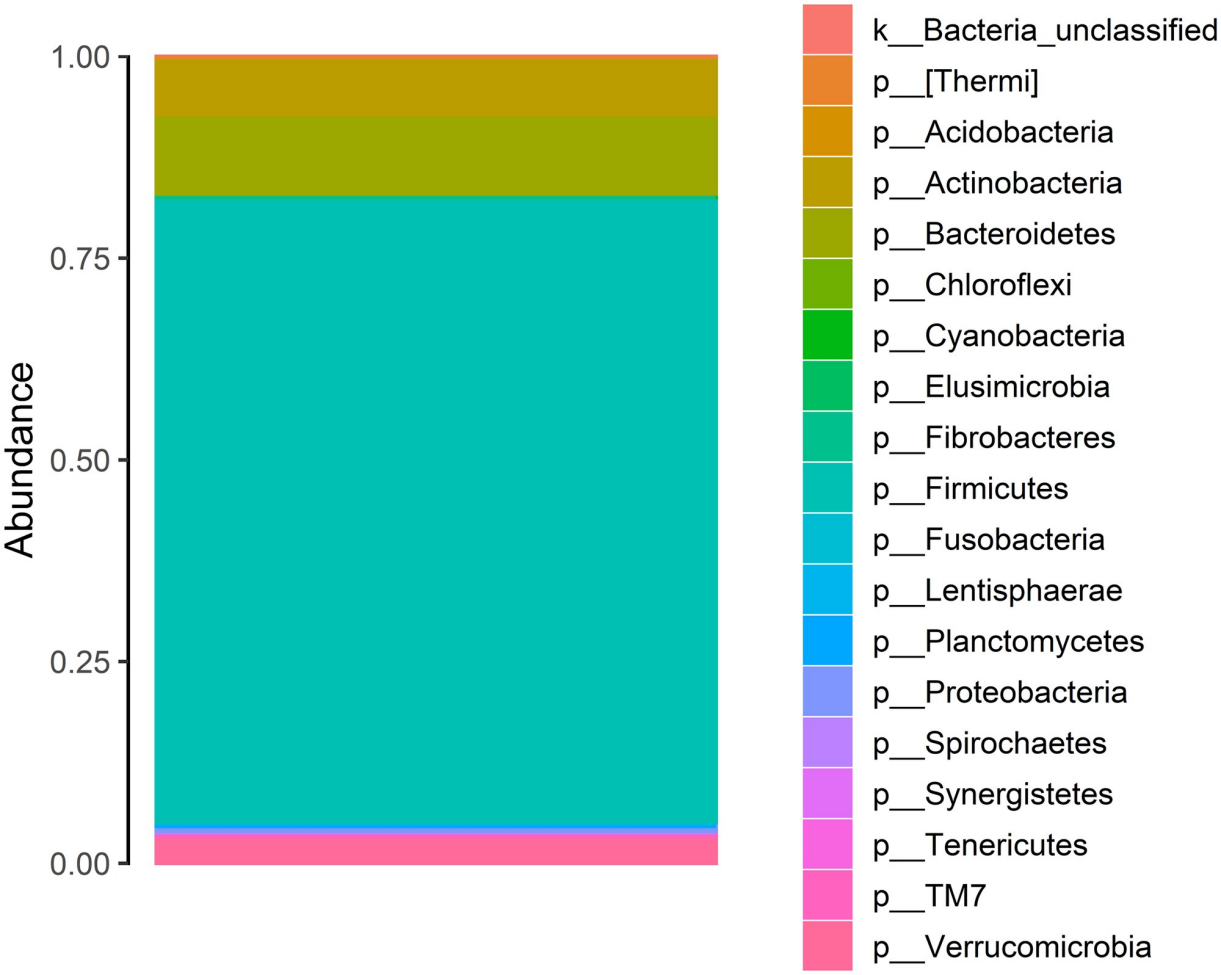
A stacked bar plot demonstrating the relative abundance of phyla in our study.

Among the 720 participants, sequencing of the V3-V4 region of the 16S rRNA gene resulted in 23,788,688 reads after filtering of chimeras, low quality reads, and sequences of incorrect length. Samples had an average of 32,632 reads. Filtered reads were assigned to 6,645 unique OTUs. After rarefaction of OTU counts to an even depth of 10,000 reads, there were 4,859 unique taxa. Six participants were removed from analysis due to low read count. After rare taxa were removed, there were 865 unique taxa. Overall, the most abundant phyla were Actinobacteria, Bacteroidetes, Firmicutes, Proteobacteria, and Verrucomicrobia (Fig 1).

On average, participants were 55 years old (SD: 16). Participants were 58% female, with 83% of participants self-identifying as White, 10% as Black, and as 3.5% Hispanic. Participants had an average body mass index of 30.7 kg/m^2^ (SD: 7.6) (Table 1).

**Table 1:** Descriptive sample characteristics, Survey of the Health of Wisconsin, 2016–2017.

Variable	Mean	SD	N	Percent
Age (years)	54.8	16.2		
Sex				
Male			305	42.3
Female			415	57.6
Race/ethnicity				
White			596	82.8
Black			75	10.4
Hispanic			25	3.5
Other			24	3.5
BMI (kg/m^2^)	30.7	7.6		
Household income per person (in 1,000 USD)	30.9	22.1		
Census category				
Urban			499	69.3
Rural			221	30.7
Education				
Some high school			44	6.1
High school			153	21.3
Some college			258	35.8
Bachelor’s			176	24.4
More than Bachelor’s			89	12.4
Smoking status				
Current			96	13.4
Former			216	29.9
Never			408	56.7
Diabetes				
Yes			113	15.7
No			607	84.3
Depression				
Yes			174	24.2
No			546	75.8
PPI use				
Yes			105	14.6
No			615	85.4
Antibiotic use				
Yes			248	34.4
No			472	65.6
Carbohydrates (g)	233.8	153.6		
Fat (g)	78.0	48.6		
Protein (g)	74.8	41.8		
Fiber (g)	19.8	11.4		
Alcohol (g)	10.4	25.9		
Average sleep duration (min/day)	512.7	79.5		
Average sedentary time (min/day)	588.8	93.0		
Average light activity (min/day)	343.8	97.9		
Physically Active				
Yes			358	49.7
No			362	50.5
Average MVPA (min/week)	195.9	172.2		
Active transportation				
Yes			148	20.5
No			572	79.5
Active transportation (min/week)	48.7	183.9		

USD, United States dollar; PPI, proton pump inhibitor; min, minutes.

We investigated several measures of physical activity, and their relationship with alpha-diversity (Table 2). We did not find strong evidence that physically active status, moderate to vigorous physical activity (MVPA) minutes per week, or any active transportation were associated with alpha-diversity. However, a one standard deviation increase in weekly minutes spent in active transportation was associated with an increase of 7.57 (95% CI: 2.55, 12.59) in Chao1’s richness and 0.04 (0.0008, 0.09) in Shannon’s diversity. Due to the compositional nature of our physical activity measures, we also adjusted for light activity rather than sedentary behavior to assess whether changing physical activity by increasing or decreasing sedentary behavior versus light activity had a differential impact on alpha-diversity. There were not substantial differences between models which adjusted for sedentary time versus those that adjusted for light activity (S2 Table).

**Table 2:** Linear mixed effects model estimates of the relationship between physical activity and alpha-diversity.

Physical activity measure	Chao1	Shannon	Inverse Simpson
	Estimate (95% CI)	Estimate (95% CI)	Estimate (95% CI)
Physically active (reference = No)	0.34 (-9.55, 10.23)	0.007 (-0.08, 0.09)	0.38 (-0.84, 1.60)
MVPA min/week (per pop. SD– 172 min.)	-1.12 (-5.79, 3.54)	-0.02 (-0.06, 0.02)	-0.24 (-0.80, 0.33)
Active transportation (reference = No)	5.86 (-3.52, 15.23)	0.03 (-0.05, 0.11)	0.24 (-0.92, 1.39)
Active transportation min/week (per pop. SD– 184 min.)	7.57*** (2.55, 12.59)	0.04** (0.0008, 0.09)	0.40 (-0.23, 1.01)

Linear mixed effects models were adjusted for age, sex, race/ethnicity, body mass index, household income per person, education, census category, smoking status, diabetes, depression, proton pump inhibitor use, antibiotic use, carbohydrate intake, protein intake, fat intake, fiber intake, alcohol intake, average sedentary time per day, average sleep duration, and sample age. Models included random intercepts to account for clustering of participants by household.

CI, confidence interval; MVPA, moderate to vigorous physical activity; min, minutes; pop, population; SD, standard deviation.

* p < 0.1.

** p < 0.05.

*** p < 0.01.

Next, we modeled several interactions in the relationship between physical activity and alpha diversity (Table 3). We found that participating in any active transportation in an urban environment was not significantly different from the overall estimate of the impact of active transportation on diversity, while participating in active transportation in a rural environment was associated with an increase of 4.69 (95% CI: 1.64, 7.73). Similarly, we found that an increase of one standard deviation of minutes in active transportation per week was associated with an increase of 2.48 (95% CI: 0.85, 4.12) among those living in a rural environment.

**Table 3:** Linear mixed effects model estimates of the relationship between physical activity and alpha-diversity, summary of models with interaction.

	Inverse Simpson	
Physical Activity Measure	Estimate (95% CI)	Estimate (95% CI)
Active transportation (reference = No)	-0.54 (-1.80, 0.73)	
Active transportation x Census category (reference = Urban)	4.69*** (1.64, 7.73)	
Active transportation min/week (per pop. SD– 184 min.)		-0.02 (-0.70, 0.65)
Active transportation min/week x Census category (reference = Urban)		2.48*** (0.85, 4.12)

Linear mixed effects models were adjusted for age, sex, race/ethnicity, body mass index, household income per person, education, census category, smoking status, diabetes, depression, proton pump inhibitor use, antibiotics use, carbohydrate intake, protein intake, fat intake, fiber intake, alcohol intake, average sedentary time per day, average sleep duration, and sample age. Models included random intercepts to account for clustering of participants by household.

CI, confidence interval; pop, population; min, minutes. SD, standard deviation.

* p < 0.1.

** p < 0.05.

*** p < 0.01.

**Fig 2:**
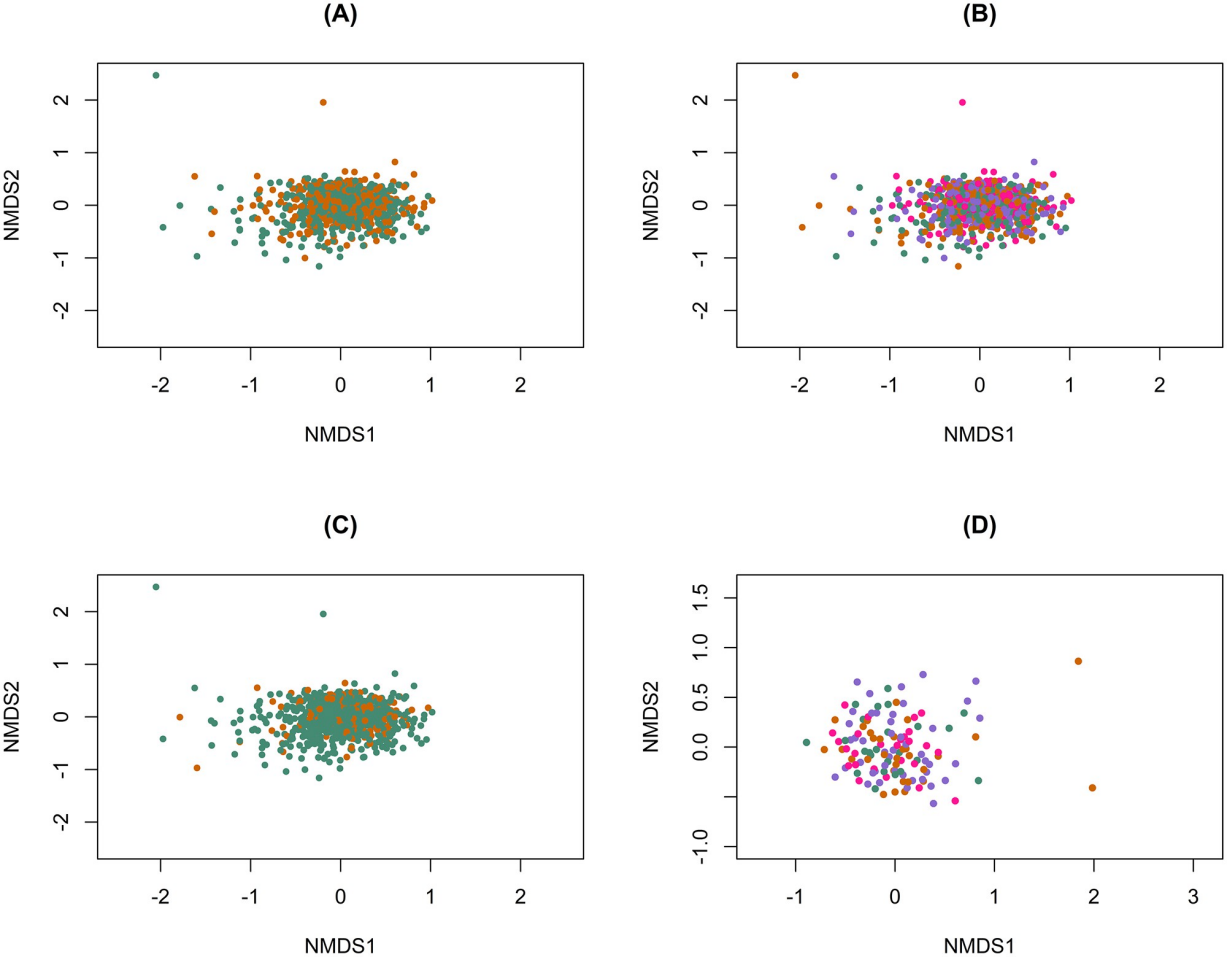
Bray-Curtis dissimilarity distances, colored by measures of physical activity. (A) Bray-Curtis dissimilarity distances, colored by physically active status. PERMANOVA R^2^ = 0.004 (*P* = 0.001). SHOW, 2016–2017. (B) Bray-Curtis dissimilarity distances, colored by quartile of MVPA minutes per week. PERMANOVA R^2^ = 0.003 (*P* = 0.001). SHOW, 2016–2017. (C) Bray-Curtis dissimilarity distances colored by engagement in any active transportation. R^2^ = 0.003 (*P* = 0.009). SHOW, 2016–2017. (D) Bray-Curtis dissimilarity distances, colored by quartile of minutes in active transportation per week, excluding participants who reported no active transportation (n = 147). PERMANOVA R^2^ = 0.004 (*P* = 0.92). SHOW, 2016–2017.

To characterize the association between microbiome composition and physical activity measures, we created non-metric multi-dimensional scaling (NMDS) plots using Bray-Curtis distance matrices colored by measures of physical activity (Fig 2). We did not observe visual clustering by the physical activity variables, but PERMANOVA tests revealed that there were differences in bacterial composition by physically active status (P = 0.001), MVPA minutes per week (P = 0.001), and engagement in active transportation (P = 0.009).

Finally, we conducted several negative binomial regressions on individual genera and family abundance (Table 4). The results presented are statistically significant (P < 0.05) before correction for multiple testing. Results did not remain significant after correction for multiple testing. We found that any active transportation was associated with an increased abundance of genus *Phascolarctobacterium* and a decreased abundance of *Clostridium* from family Erysipelotrichaceae (P = 0.047 and 0.03, respectively). Being physically active and increased weekly minutes of MVPA was associated with an increase in the abundance of bacteria from the family Erysipelotrichaceae (P = 0.01). Increased weekly MVPA minutes were also associated with an increase in an unknown family and genus from the order Clostridiales (P = 0.03). Increased minutes in active transportation was associated with a decrease in the abundance of bacteria from the family Clostridiaceae (P = 0.03).

**Table 4:** Results of negative binomial models, before and after adjustment for multiple testing.

	**Genus**	**Direction**	**P**	**P**_**fdr**_
Active transportation	Phascolarctobacterium	↑	0.047	0.6
	Clostridium (family: Erysipelotrichaceae)	↓	0.03	0.6
MVPA min/week	Unknown (order: Clostridiales)	↑	0.03	0.7
	**Family**	**Direction**	**P**	**P**_**fdr**_
Physically active	Erysipelotrichaceae	**↑**	0.01	0.1
MVPA min/week	Erysipelotrichaceae	**↑**	0.01	0.1
	Unknown (order: Clostridiales)	**↑**	0.03	0.2
Minutes in active transportation	Clostridiaceae	**↓**	0.03	0.3

Models adjusted for: age, sex, race/ethnicity, body mass index, household income per person, education, census category, smoking status, diabetes, depression, proton pump inhibitor use, antibiotic use, carbohydrate intake, protein intake, fat intake, fiber intake, alcohol intake, average sedentary time per day, average sleep duration, and sample age. fdr, false discovery rate; MVPA, moderate to vigorous physical activity; min, minutes.

## Discussion

Globally, insufficient physical activity is a leading cause of preventable disease, particularly in Western countries such as the United States [60]. In this study, we sought to understand the effects of physical activity on the gut microbiome, including understanding outdoor physical activity, which has recently increased in popularity in the US [61]. This large population-based analysis adds to the growing body of evidence that the gut microbiome may be linked with physical activity. Within this geographically diverse study population, we found that increased weekly minutes in active transportation were associated with increased alpha-diversity of the gut microbiota. Overall, we saw mixed results, consistent with previous study findings. In particular, we did not find strong evidence that being physically active or increased weekly time in MVPA was associated with changes in alpha diversity, while increased minutes in active transportation were associated with increased alpha-diversity. This latter finding is consistent with previous studies that also found a relationship between alpha-diversity and physical activity [23, 29].

We found that the abundance of an unknown family from order Clostridiales was associated with increased weekly MVPA minutes. Clostridiales is a beneficial butyrate-producing taxon which Estaki et al. found to be associated with increased physical fitness [29]. Butyrate has previously been associated with improved gut-barrier integrity [62], and has anti-oxidant, anti-carcinogenic, and anti-inflammatory properties [63]. The abundance of family Erysipelotrichaceae in the gut differed by weekly MVPA minutes. Previous studies have found that Erysipelotrichaceae plays a role in lipid metabolism [64]. Increased abundance of *Phascolarctobacterium* was associated with active transportation in our study. Previous research has found that higher abundance of *Phascolactobacterium* was associated with insulin sensitivity [65]. Given the well-established associations between physical activity and metabolic health in general, these findings are consistent and point to a potential pathway by which the gut microbiota may be linked to physical activity and other well established health benefits. Further research using metabolomic and/or functional metagenomics may offer additional insights.

While our findings regarding genera or families associated with physical activity measures were not statistically significant after correction for multiple testing, we present P-values both before and after adjustment while noting that the Benjamini-Hochberg procedure may be an overadjustment because the Benjamini-Hochberg procedure assumes the independence of tests, and gut microbial abundances are correlated [66, 67].

This study is unique in its examination of active transportation, and its findings regarding the interaction between census category and active transportation suggest that spending time outdoors may also be beneficial beyond the benefits of physical activity. Because active transportation necessitates completing physical activity outdoors, while MVPA does not, differences seen in the associations may be due to the added outdoor exposure. A previous study found that time spent outside without physical activity can alter the gut microbiome of children [68], making it plausible that there are additional changes in the gut microbiota specific to exercising outdoors, beyond the activity itself. The interactions we observed between active transportation and rural living support this idea, as the outdoor component of active transportation is likely to be different in those contexts compared to more urban settings. For example, outdoor physical activity in an urban setting may expose individuals to increased levels of traffic-related air pollution, which has been shown to affect the microbiome [69]. However, especially because minutes in active transportation are self-reported, it may be that the relationship between minutes spent in active transportation and the gut microbiome is positively confounded by some additional unmeasured variable. Further investigation into the potential benefits of completing physical activity outdoors is needed to better understand this relationship.

Despite numerous strengths, including large sample size, and objectively measured physical activity, as well as the ability to control for several potential confounders, there are a few limitations to note. First, the study relies on the use of 16S rRNA data, from which we could not identify taxonomy more specific than the genus-level; future studies should leverage species-level taxonomic information and pathway analysis to better understand the mechanisms driving these relationships. Additionally, this study conducted accelerometer measurement for one week. Although 3–5 days of measurement has been shown to be sufficient to estimate typical physical activity and sedentary behavior [70], we did not collect data about habitual physical activity or exercise regimens. Aside from characterizing active transportation, we do not have information on the types of activity participants completed during measurement. Future studies should consider augmenting accelerometry data with details on the types of physical activities performed for a more comprehensive view of physical activity. This study also used actigraphy to estimate sleep durations. Typically, polysomnography would be preferred as the gold standard, but studies have found that actigraphy is an acceptable substitute in healthy adults [71, 72], and the Cole-Kripe algorithm described in this study has been shown to have 83.86% agreement with polysomnography [47]. Furthermore, there may be some residual confounding by several measures. Errors in dietary estimates can be inherently biased and resemble a ‘flattened slope’ wherein those with high intakes tend to under-report and those with low intake tend to over-report [73]. This type of measurement error would induce bias in estimates of the association between dietary variables and the microbiome. Another important potential source of confounding that we were unable to adjust for was environmental exposures. Previous research has found that the gut microbiome can be impacted by exposure to lead [39] and other heavy metals [74], and air pollution [69]. These exposures were not included in our analysis. To the extent that active transportation in an urban environment corresponds with exposure to air pollution, air pollution may be an important confounder of these findings. Finally, while we adjusted for diabetes diagnosis by a physician, we were not able to explicitly adjust for use of anti-diabetic medications. Participants who did not report a diabetes diagnosis, but who were using anti-diabetic medications may have incorrectly been classified as non-diabetics, resulting in the potential for residual confounding.

## Conclusions

This study provides some evidence that physical activity is associated with increased abundance of health-promoting gut microbes including Clostridiales. Additionally, we found preliminary evidence that outdoor physical activity has additional benefit to the gut microbiome, compared to physical activity alone. Future research should further evaluate the effects of physical activity on the gut microbiome in comparison with sedentary time spent outdoors and physical activity completed indoors.

## Supporting information

S1 Fig Summary of missing accelerometery data. Light blue represents intervals with missing data.
(TIF)

S1 Table Bivariate linear regression results for possible *a priori* confounders of the relationship between physical activity and the microbiome. Bold cells indicate significance at P < 0.05. MVPA, moderate to vigorous physical activity; CI, confidence interval; Ref, reference; USD, United States dollars; PPI, proton pump inhibitor; NSAID, non-steroidal anti-inflammatory drugs; Avg, average; min, minutes.
(DOCX)

S2 Table Linear mixed effects model estimates of the association between physical activity and alpha-diversity. Linear mixed effects models were adjusted for age, sexr, race/ethnicity, body mass index, household income per person, education, census category, smoking status, diabetes, depression, proton pump inhibitor use, antibiotic use carbohydrate intake, fat intake, fiber intake, alcohol intake, average light activity per day, average sleep duration, and sample age. Models included a random intercept to account for clustering of participants by household. CI, confidence interval; MVPA, moderate to vigorous physical activity; pop, population; SD, standard deviation. * p < 0.1; ** p < 0.05; *** p < 0.01.
(DOCX)
