# Supplementary figures and images for: Assessing the relationship between physical activity and the gut microbiome in a large, population-based sample of Wisconsin adults
Source: PLoS One. 2022 Oct 26;17(10):e0276684. doi: 10.1371/journal.pone.0276684 (PMC9605031; doi:10.1371/journal.pone.0276684)

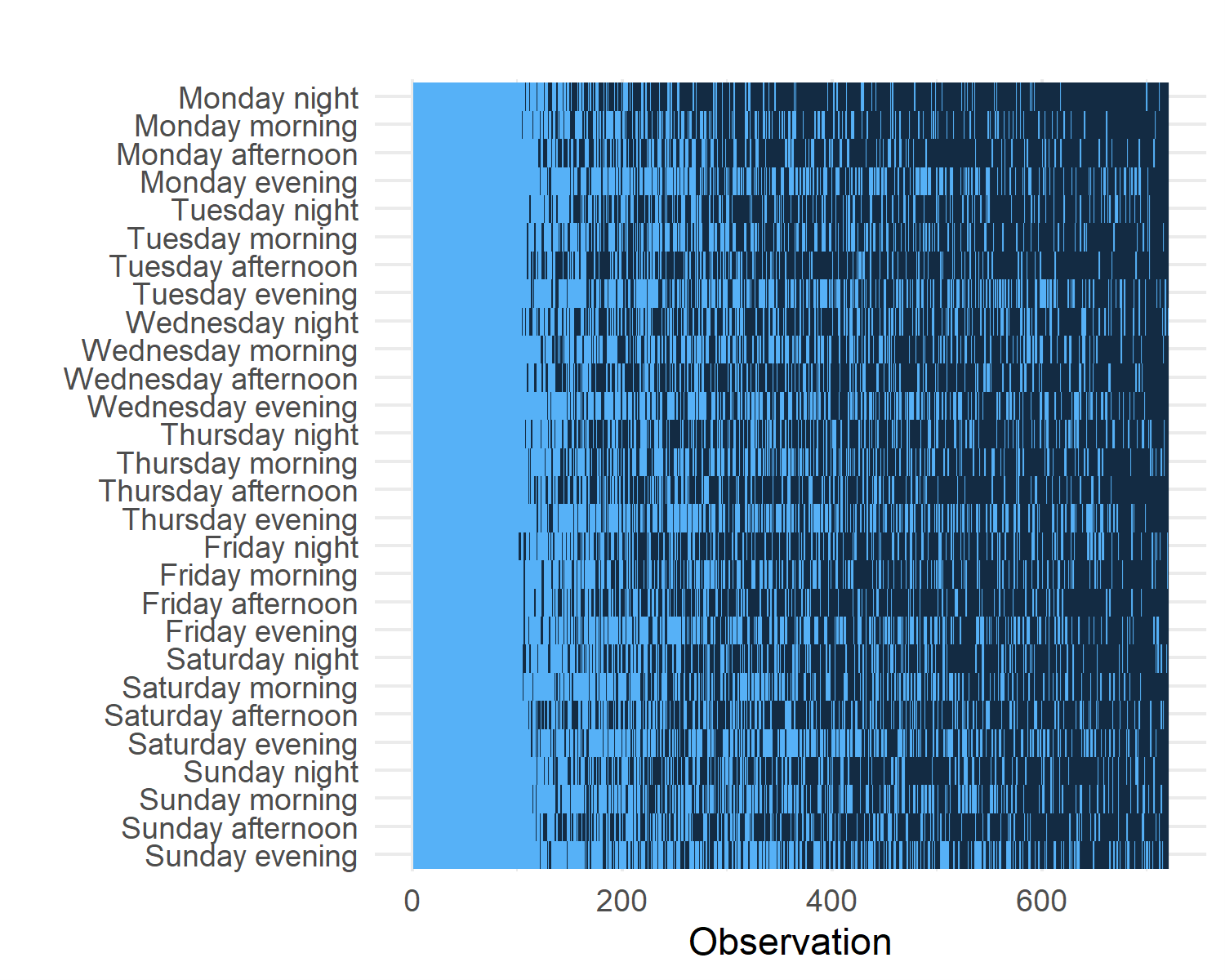

Supplement: S1 Fig — Light blue represents intervals with missing data. (TIF) [file pone.0276684.s001.tif]
