# Supplementary material for: Assessing the relationship between physical activity and the gut microbiome in a large, population-based sample of Wisconsin adults
Source: PLoS One. 2022 Oct 26;17(10):e0276684. doi: 10.1371/journal.pone.0276684 (PMC9605031; doi:10.1371/journal.pone.0276684)
Supplement: S1 Table — Bold cells indicate significance at P < 0.05. MVPA, moderate to vigorous physical activity; CI, confidence interval; Ref, reference; USD, United States dollars; PPI, proton pump inhibitor; NSAID, non-steroidal anti-inflammatory drugs; Avg, average; min, minutes. (DOCX) [file pone.0276684.s002.docx]

| Variable | Physically active  Estimate (95% CI) | Average MVPA  Estimate (95% CI) | Active transportation  Estimate (95% CI) | Minutes in active transportation  Estimate (95% CI) |
| --- | --- | --- | --- | --- |
| Age (years) | **-0.04 (-0.06, -0.03)** | **-0.02 (-0.02, 0.01)** | **-0.02 (-0.03, -0.004)** | -0.002 (-0.004, 0.003) |
| Sex (Ref = Female) | 0.53 (0.12, 0.93) | 0.28 (0.13, 0.44) | 0.17 (-0.22, 0.57) | 0.16 (0.06, 0.27) |
| Race/Ethnicity  White  Black  Hispanic  Other | Ref  -0.05 (-0.83, 0.72)  0.09 (-1.04, 1.23)  -0.32 (-1.41, 0.77) | Ref  0.04 (-0.24, 0.32)  -0.13 (-0.24, 0.32)  0.15 (-0.29, 0.58) | Ref  **1.19 (0.55, 1.84)**  0.62 (-0.41, 1.64)  0.95 (-0.06, 1.97) | Ref  **0.24 (0.06, 0.43)**  -0.04 (-0.34, 0.26)  0.17 (-0.14, 0.47) |
| Body Mass Index (kg/m^2^) | **-0.05 (-0.09, -0.02)** | **-0.02 (-0.03, 0.01)** | **-0.07 (-0.10, -0.03)** | **-0.01 (-0.14, 0.005)** |
| Household income per person (in 1,000 USD) | **0.23 (0.009, 0.45)** | **0.08 (-0.008, 0.17)** | 0.03 (-0.17, 0.24) | -0.01 (-0.07, 0.04) |
| Census category  Urban  Rural | Ref  **-0.56 (-1.00, -0.13)** | Ref  **-0.20 (-0.39, -0.02)** | Ref  -**1.26 (-1.81, -0.72)** | Ref  **-0.17 (-0.29, -0.04)** |
| Education  Some high school  High school  Some college  Bachelor’s  More than Bachelor’s | 0.64 (-0.03, 1.31)  0.14 (-0.40, 0.69)  -0.12 (-0.60, 0.35)  Ref  -0.14 (-0.50, 0.22) | 0.25 (-0.01, 0.51)  0.04 (-0.19, 0.27)  -0.03 (-0.22, 0.16)  Ref  -0.11 (-0.27, 0.05) | 0.60 (-0.08, 1.27)  0.25 (-0.36, 0.85)  0.20 (-0.30, 0.70)  Ref  0.06 (-0.34, 0.46) | -0.02 (-0.20, 0.16)  -0.02 (-0.17, 0.14)  0.05 (-0.08, 0.18)  Ref  0.04 (-0.07, 0.14) |
| Live or work on a farm (Ref = No) | 0.24 (-0.50, 0.98) | 0.09 (-0.24, 0.41) | -0.88 (-1.88, 0.12) | -0.01 (-0.23, 0.20) |
| Smoking status  Current  Former  Never | -0.61 (-1.24, 0.03)  **-0.51 (-0.95, -0.08)**  Ref | **-0.25 (-0.49, -0.009)**  **-0.27 (-0.46, -0.07)**  Ref | -0.01 (-0.64, 0.62)  -0.03 (-0.50, 0.44)  Ref | 0.13 (-0.04, 0.29)  0.04 (-0.08, 0.17)  Ref |
| Diabetes (Ref = No) | **-0.96 (-1.53, -0.38)** | **-0.37 (-0.59, -0.15)** | -0.76 (-1.45, -0.06) | -0.04 (-0.19, 0.12) |
| Nursing home/inpatient  (Ref = No) | -0.45 (-1.36, 0.47) | -0.002 (-0.33, 0.33) | -0.35 (-1.33, 0.63) | -0.09 (-0.33, 0.15) |
| Depression (Ref = No) | -0.53 (-1.06, 0.0008) | -0.17 (-0.36, 0.02) | -0.20 (-0.69, 0.29) | -0.07 (-0.20, 0.06) |
| PPI Use (Ref = No) | **-0.67 (-1.24, -0.09)** | **-0.34 (-0.56, -0.11)** | -0.49 (-1.14, 0.15) | -0.05 (-0.51, 0.10) |
| Taking antibiotics (Ref = No) | **-0.42 (-0.82, -0.01)** | **-0.20 (-0.38, -0.03)** | -0.38 (-0.85, 0.09) | -0.11 (-0.23, 0.01) |
| Total antibiotic days | -0.02 (-0.06, 0.02) | -0.007 (-0.02, 0.009) | -0.05 (-0.10, 0.007) | -0.006 (-0.02, 0.003) |
| NSAID use (Ref = No) | -0.17 (-0.61, 0.26) | -0.09 (-0.27, 0.09) | -0.29 (-0.74, 0.16) | -0.02 (-0.02, 0.003) |
| Carbohydrates (g) | 0.001 (-0.0003, 0.003) | **0.0008 (0.0003, 0.001)** | **0.002 (0.0003, 0.003)** | **0.0006 (0.0001, 0.001)** |
| Protein (g) | **0.005 (0.0001, 0.01)** | **0.004 (0.001, 0.006)** | **0.006 (0.001, 0.01)** | **0.002 (0.0008, 0.004)** |
| Fat (g) | 0.003 (-0.0008, 0.008) | **0.003 (0.001, 0.004)** | **0.005 (0.0008, 0.01)** | **0.002 (0.0009, 0.004)** |
| Fiber (g) | 0.02 (-0.0006, 0.04) | **0.01 (0.004, 0.02)** | **0.03 (0.01, 0.05)** | **0.007 (0.001, 0.01)** |
| Alcohol (g) | 0.006 (-0.002, 0.01) | 0.003 (-0.00003, 0.006) | 0.005 (-0.002, 0.01) | 0.0008 (-0.001, 0.003) |
| Avg. sleep duration (min/day) | **-0.005 (-0.009, -0.0005)** | **-0.002 (-0.005, -0.0005)** | -0.00006 (-0.003, 0.003) | 0.00005 (-0.0007, 0.0008) |
| Avg. sedentary time (min/day) | **-0.005 (-0.009, -0.002)** | **-0.002 (-0.004, -0.0009)** | **-0.003 (-0.006, -0.0008)** | **-0.0007 (-0.001, -0.00006)** |
| Avg. light activity (min/day) | **0.007 (0.004, 0.01)** | **0.004 (0.003, 0.005)** | 0.002 (-0.0004, 0.4) | 0.0004 (-0.0002, 0.001) |
