# Supplementary material for: Assessing the relationship between physical activity and the gut microbiome in a large, population-based sample of Wisconsin adults
Source: PLoS One. 2022 Oct 26;17(10):e0276684. doi: 10.1371/journal.pone.0276684 (PMC9605031; doi:10.1371/journal.pone.0276684)
Supplement: S2 Table — Linear mixed effects models were adjusted for age, sexr, race/ethnicity, body mass index, household income per person, education, census category, smoking status, diabetes, depression, proton pump inhibitor use, antibiotic use carbohydrate intake, fat intake, fiber intake, alcohol intake, average light activity per day, average sleep duration, and sample age. Models included a random intercept to account for clustering of participants by household. CI, confidence interval; MVPA, moderate to vigorous physical activity; pop, population; SD, standard deviation. * p < 0.1; ** p < 0.05; *** p < 0.01. (DOCX) [file pone.0276684.s003.docx]

|  | **Chao1** | **Shannon** | **Inverse Simpson** |
| --- | --- | --- | --- |
| **Physical activity measure** | **Estimate (95% CI)** | **Estimate (95% CI)** | **Estimate (95% CI)** |
| Physically active (reference = No) | 1.00 (-9.09, 11.10) | 0.007 (-0.08, 0.09) | 0.30 (-0.94, 1.54) |
| MVPA min/week  (per pop. SD – 172 min) | -0.76 (-5.47, 3.95) | -0.02 (-0.64, 0.02) | -0.30 (-0.89, 0.27) |
| Active transportation (reference = No) | 5.94 (-3.42, 15.29) | 0.03 (-0.05, 0.11) | 0.25 (-0.90, 1.40) |
| Minutes active transportation per week  (per pop. SD – 184 min) | 7.59^***^ (2.59, 12.60) | 0.04^**^ (0.001, 0.09) | 0.39 (-0.23, 1.01) |
